# Prevalence of and risk factors for sarcopenia in community‐dwelling people: The Vietnam Osteoporosis Study

**DOI:** 10.1002/jcsm.13383

**Published:** 2023-12-25

**Authors:** Duy K. Hoang, Minh C. Doan, Nhan M. Le, Huy G. Nguyen, Lan T. Ho‐Pham, Tuan V. Nguyen

**Affiliations:** ^1^ University of Technology Sydney Sydney New South Wales Australia; ^2^ Biomedical Research Center Pham Ngoc Thach University of Medicine Ho Chi Minh City Vietnam; ^3^ Saigon Precision Medicine Research Center Ho Chi Minh City Vietnam; ^4^ Bone and Muscle Research Group Ton Duc Thang University Ho Chi Minh City Vietnam; ^5^ School of Population Health UNSW Sydney Sydney New South Wales Australia

**Keywords:** appendicular skeletal muscle mass, attributable risk, muscle strength, prevalence, risk factor, sarcopenia, Vietnam Osteoporosis Study

## Abstract

**Background:**

Sarcopenia is a geriatric disease characterized by the progressive and generalized loss of skeletal lean mass and strength with age. The prevalence of sarcopenia in the Vietnamese population is unknown. This study sought to estimate the prevalence of and risk factors for sarcopenia among community‐dwelling individuals in Vietnam.

**Methods:**

This cross‐sectional study is part of the ongoing Vietnam Osteoporosis Study project. The study involved 1308 women and 591 men aged 50 years and older as at 2015 (study entry). Whole‐body dual‐energy X‐ray absorptiometry was used to measure the appendicular skeletal lean mass. Anthropometric and clinical data were collected using a structured questionnaire. Sarcopenia was defined according to the criteria proposed by the Asian Working Group for Sarcopenia in 2019. Logistic regression analysis was used to determine the association between potential risk factors and sarcopenia.

**Results:**

The prevalence of sarcopenia in women and men was 14% (*n* = 183) and 16% (*n* = 83), respectively. Age (odds ratio [OR] per 10 years = 1.37; 95% confidence interval [CI] 1.26–1.48) and being underweight (OR = 1.61; 95% CI 1.00–2.58) were independently associated with increased risk of sarcopenia. The combination of low physical activity, being underweight and advancing age accounted for ~27% of sarcopenic patients. However, most of the attributable fraction was due to ageing.

**Conclusions:**

Sarcopenia is common in community‐dwelling Vietnamese adults, particularly those with advancing age, who are underweight and with low physical activity.

## Introduction

Sarcopenia is a geriatric disease characterized by a reduction in lean mass and impairment of muscle function.^1^ Sarcopenia can be a severe disease associated with an increased risk of falls, functional impairment and mortality.^2^ A recent meta‐analysis study showed that sarcopenia was associated with an increased risk of falls with a relative risk ranging from 1.60 to 1.9.^3^ More importantly, people with sarcopenia have a greater mortality,^4^, ^5^ and the risk was particularly pronounced in cancer patients.^6^ Data from the National Health and Nutrition Examination Survey indicated that sarcopenic individuals also had higher odds of hospitalization, and the cost of hospitalization was projected to be $40.4 billion in 2014.^7^ With the ongoing ageing population being taken place worldwide, the prevalence of sarcopenia and its consequences are expected to become a significant public health burden in the future.

Although sarcopenia has recently received considerable attention from the medical research community, it is difficult to estimate its prevalence due to largely a lack of consensus definition. In 2010, the European Working Group on Sarcopenia in Older People (EWGSOP) proposed a series of diagnostic criteria based on lean mass and muscle function for sarcopenia.^8^ In 2011, the International Working Group on Sarcopenia (IWGS) published another set of criteria, including gait speed and appendicular skeletal muscle mass (ASM), for the diagnosis of sarcopenia.^9^ However, the EWGSOP and IWGS criteria were based on data obtained from Caucasian populations whose body size and lean mass are systematically different from Asian populations. Therefore, in 2019, the Asian Working Group for Sarcopenia (AWGS) proposed a set of different criteria for the diagnosis of sarcopenia in Asians.^10^ The AWGS criteria are based on lean mass and muscle strength and/or physical performance.

As a result, prevalence estimates of sarcopenia are highly variable between populations, with point estimates ranging between 6% and 22%.^11^ In Asia, the prevalence of sarcopenia among community‐dwelling people aged 65 and over is ~11% in men and 4% in women.^12^ Until now, there is, however, no population‐based study of sarcopenia in Vietnam.

Vietnam is a dynamic country with a population of ~100 million, of which ~8% aged 65 years and older.^13^ Economic development in Vietnam has generated profound changes in lifestyle and impact on population health, with non‐communicable diseases becoming a major public health concern.^14^ The prevalence of and risk factors for sarcopenia in the general community remain undocumented in Vietnam. Therefore, this study sought to estimate the prevalence of sarcopenia and its associated risk factors in the Vietnamese population.

## Study design and methods

This study was part of the Vietnam Osteoporosis Study (VOS) project initiated in mid‐2015. VOS was designed as a longitudinal cohort study in which ~4200 individuals were randomly sampled from Ho Chi Minh City, Vietnam.^15^ The data collection was conducted between 2015 and 2017. We used two approaches to recruit participants. In the first approach, we contacted community organizations to solicit a list of members, and from the list, we ran a computer program to randomly selected individuals who met the age and sex criteria. A letter was then sent to the selected individuals to invite them and their family members to participate in the study. In the second approach, we recruited participants via television, the Internet and flyers in universities. The flyers described (in Vietnamese) the study's purposes, procedures and benefits of participants. Individuals who agreed to participate in the study were then transported to the Bone and Muscle Research Laboratory at the Ton Duc Thang University for clinical assessment and evaluation. The participants did not receive any financial incentive, but they received a free health check‐up and lipid analyses. The inclusion criteria were broad: Men and women aged between 18 years and older agreed to participate in the study. The current study included only individuals aged 50 years and older. We excluded individuals deemed to have impaired cognitive function, unwilling to give informed consent or physically unable to complete clinical tests. The study's procedure and protocol were approved by the Ethics Committee of the People's Hospital 115, Ho Chi Minh City. All participants gave written informed consent. Participants could withdraw from the study at any time without giving reasons.

Height and body weight were measured by an electronic portable, wall‐mounted stadiometer (Seca Model 769; Seca Corp., CA, USA) without shoes, hats, ornaments or heavy layers of clothing. Body mass index (BMI) was calculated as weight (kg) divided by the square of height (kg/m^2^).

Physical activity was ascertained using the Global Physical Activity Questionnaire (GPAQ).^16^ The GPAQ has questions on the type and intensity of exercise and walking in the last 7 days. The metabolic task equivalent (MET) was determined by the reported weekly minutes spent by the corresponding MET score of each category (8 for vigorous activities, 4 for moderate activities and 3.3 for walking). The overall score was then calculated by summing the score of the three types of activity in MET min/week. Physical activity levels were categorized as high (≥1200 MET min/week), medium (600 to <1200 MET min/week) or low (≤600 MET min/week).

Lean mass and fat mass were measured by dual‐energy X‐ray absorptiometry (DXA) using a Hologic Horizon densitometer (Hologic Corp., Bedford, MA, USA) with a standard adult's whole‐body scan mode. The Hologic software Version 12.6 was used to analyse lean mass and fat mass (in kg). The densitometer was standardized by a Hologic‐designed whole‐body phantom. The phantom includes six white high‐density polyethylene rectangles, and a polyvinyl chloride sheet is bonded to a high‐density polyethylene rectangle to mimic lean mass. Areal bone mineral density (BMD) was measured at the lumbar spine, femoral neck, total hip and whole body using a Hologic Horizon (Hologic Corp., Bedford, MA, USA). For the lumbar spine, we measured BMD from L2 to L4. The densitometer was standardized by phantom before each measurement. The measurement was done by a qualified radiology technologist. Based on 20 individuals, the coefficient of variation in BMD at our lab was 1.5% for the lumbar spine and 1.7% for the hip. Fat mass and lean mass were derived from the whole‐body scan. ASM was determined as the sum of lean mass in both the arms and legs. The ASM was then adjusted for height as follows: ASMi = ASM/height squared, with the unit being kg/m^2^.

Handgrip strength measurement was performed using a Jamar dynamometer. The coefficient of reliability was 0.91. Participants were advised to sit upright with their shoulder adducted, elbow flexed to 90°, and wrist and forearm neutral. The technician places the dynamometer in the client's hand while gently setting up the dynamometer's base and instructing the client to squeeze as strongly as possible. Grip force should be applied smoothly, without rapid jerking motion. The wrist extends during the test. The measurement was carried out twice on the right and twice on the left hand. The largest value was chosen for the analysis.

### Definition of sarcopenia

In this study, sarcopenia was defined as low lean mass and low grip strength, using cut points suggested by AWGS. According to this classification, low lean mass was defined as ASMi < 7.0 kg/m^2^ in men and ASMi < 5.4 kg/m^2^ in women and cut points for handgrip strength <28 kg in men and <18 kg in women.^10^

### Data analysis

The prevalence of sarcopenia was estimated by dividing the number of individuals with sarcopenia by the total sample size, stratified by gender and age group. The 95% confidence interval (CI) of prevalence was determined using the ‘binconf’ function within the R package of ‘Hmisc’. We used the logistic regression model to ascertain the magnitude of the association between risk factors and sarcopenia. The risk factors considered in this study were gender, age, BMI and physical activity. All analyses were conducted using the R statistical environment Version 4.2.2.

We further conducted an analysis of population attributable risk fraction (PAR). This fraction is conceptually defined as the proportion of sarcopenia that can be reduced if exposure to a risk factor was reduced to an ideal exposure. For a single risk factor, PAR is estimated as $P\left(\text{OR}-1\right)/\left(1+P\left(\text{OR}-1\right)\right)$, where *P* is the proportion of the population exposed to the risk factor and *OR* is the odds ratio associated with the exposure. When there are multiple risk factors, we used the heuristic formula to estimate PAR.^17^

## Results

The study included 1899 (1308 women and 591 men) aged 50 years and older. The average age of participants was 60 years (SD 7.7). As expected, men had greater body weight and stature than women, but there was no significant difference in BMI between genders. Whole‐body lean mass and grip strength in men, on average, were 1.5 standard deviation greater than women (*Table* *1*).

**TABLE 1:** Anthropometric characteristics of 591 men and 1308 women in the study sample

	Men (*n* = 591)	Women (*n* = 1308)	*P* value
Age (years)	60.1 (7.69)	60.2 (7.66)	0.639
Weight (kg)	61.5 (9.53)	54.0 (8.57)	**<0.001**
Height (cm)	163 (5.61)	152 (5.32)	**<0.001**
Body mass index (kg/m^2^)	23.3 (3.20)	23.4 (3.34)	0.174
Grip strength	33.1 (8.14)	20.9 (5.73)	**<0.001**
Lean mass (kg)	42.6 (5.32)	30.8 (3.96)	**<0.001**
Appendicular skeletal muscle mass (kg)	18.8 (2.69)	12.7 (1.94)	**<0.001**
Low physical activity, *n* (%)	188 (30.5)	466 (34.5)	0.086

*Note*: Data are presented as mean (standard deviation) for continuous variables and *n* (%) for categorical variables. Bold emphasis indicates the significant difference between men and women.

**FIGURE 1:**
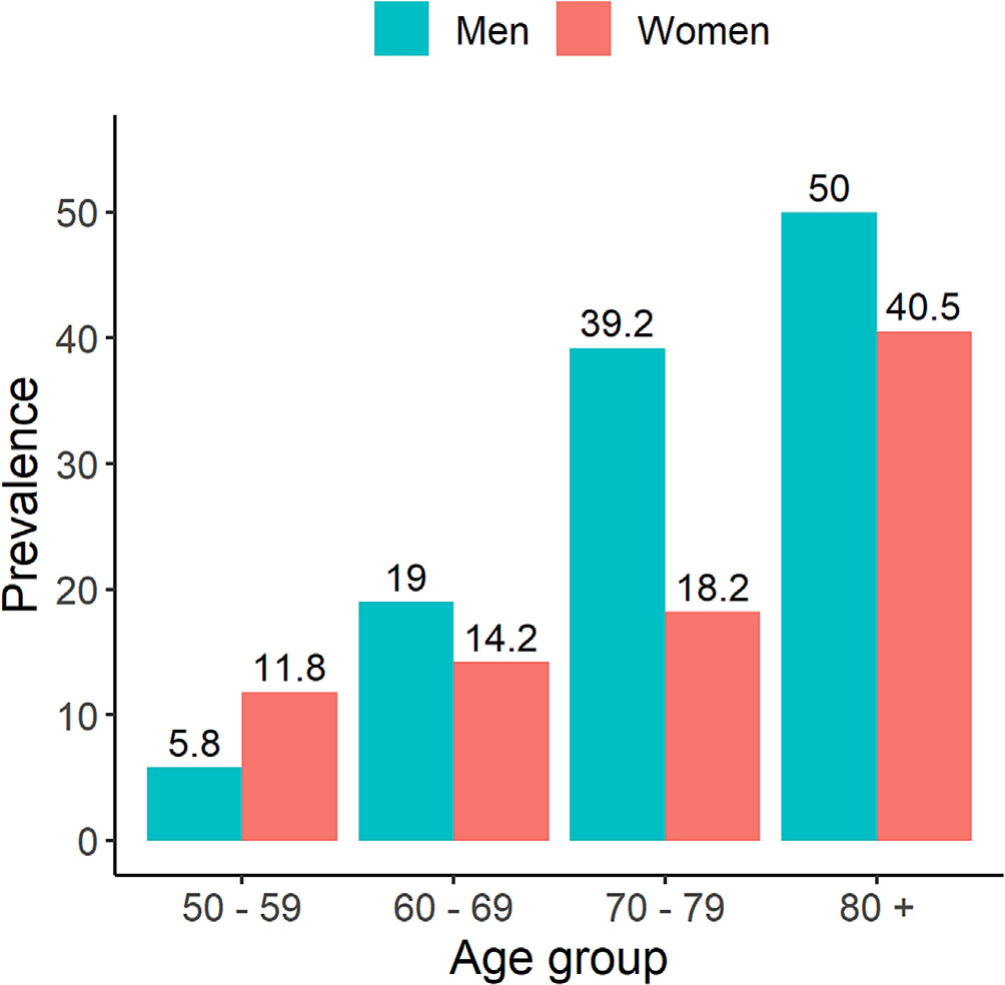
Prevalence of sarcopenia stratified by gender and age group.

Using the criteria of BMI > 25, ~32% of non‐sarcopenic individuals (*n* = 517) and 6.8% (*n* = 18) of sarcopenic individuals were classified as ‘obese’. In either gender, there was a negative correlation between ASM and grip strength with advancing age (*Figure* *1*).

### Prevalence of sarcopenia

**FIGURE 2:**
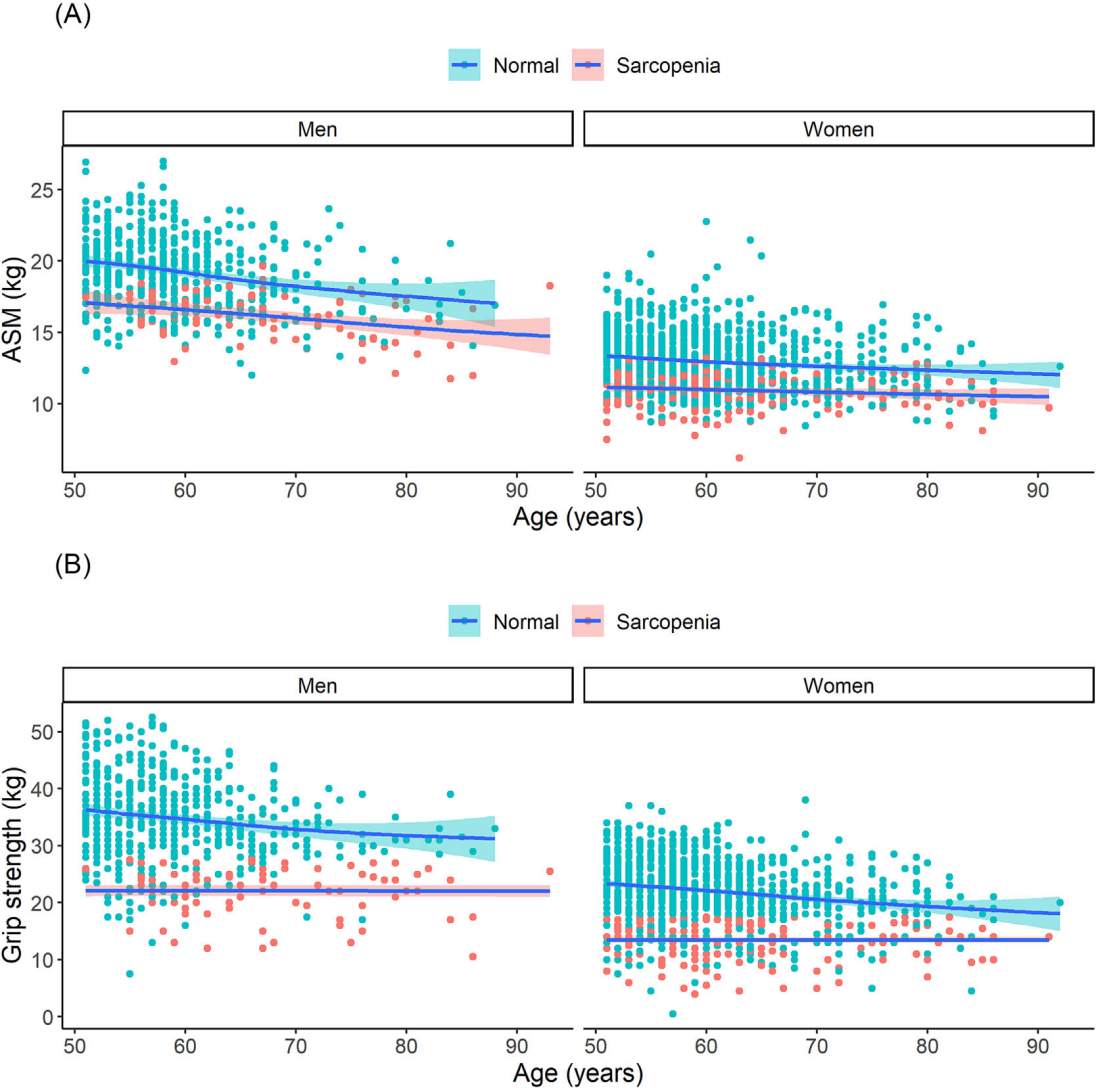
Association between age and appendicular skeletal muscle mass (ASM) (A) and grip strength (B) stratified by gender and sarcopenia.

Based on the AWGS 2019 criteria, 266 individuals had sarcopenia, making the prevalence of sarcopenia 14% (95% CI 12.1–18.2). There was no statistically significant difference in sarcopenia prevalence between women (14%; 95% CI 12.2–16.0) and men (14%; 95% CI 11.3–17.1). More importantly, the prevalence of sarcopenia increased with advancing age. Among those aged 50–59 years, the prevalence of sarcopenia was 5.8% in men, and this prevalence was increased to 50% among those aged 80 years and older; in women, this prevalence increased from 12% to 40% (*Figure* *2*).

### Risk factors for sarcopenia

In multivariable logistic regression, age, weight and physical activity were independently associated with sarcopenia. Each 10‐year increase in age was associated with 87% increase in the odds of sarcopenia (adjusted odds ratio [aOR] = 1.87; 95% CI 1.60–2.19). Moreover, compared with normal‐weight individuals, those with being overweight had lower odds of sarcopenia (aOR = 0.25; 95% CI 0.18–0.34). On the other hand, being underweight had a 61% increase in the odds of sarcopenia (aOR = 1.61; 95% CI 1.00–2.58). Moreover, high and medium physical activity was also significantly associated with a decreased risk of sarcopenia (aOR = 0.72; 95% CI 0.55–0.96) (*Table* *2*).

**TABLE 2:** Risk factors for sarcopenia: Logistic regression analysis

	Odds ratio (95% CI)	*P* value
Age (+10 years)	1.87 (1.60–2.19)	<0.001
BMI group		
Normal	1.00	
Underweight	1.61 (1.00–2.58)	0.049
Overweight	0.25 (0.18–0.34)	<0.001
Physical activity		
Low physical activity	1.00	
High + medium physical activity	0.72 (0.55–0.96)	0.025

Abbreviations: BMI, body mass index; CI, confidence interval.

### Population attributable fraction analysis

*Table* *3* shows the estimates of population attributable risk for the three risk factors identified above. In this analysis, we dichotomize age into two groups: 70 years and older or <70 years. Fifty‐seven per cent of individuals in the study sample had none of the three factors (i.e., ‘control group’). Approximately 5% of the study sample were classified as having low physical activity and advancing age, and the odds of sarcopenia among these individuals were increased by 2.7‐fold compared with the control group. The proportion of sarcopenia attributed to low physical activity and advancing age was estimated to be 8.3%. In total, the three risk factors accounted for 26.5% of cases of sarcopenia.

**TABLE 3:** Proportion of sarcopenia attributable to physical activity, being underweight and advancing age

Low PA	Underweight	Age ≥70	Prevalence	Odds ratio	Population attributable fraction (%)
No	No	No	0.57	0.44 (0.35–0.55)	0
Yes	No	No	0.26	1.01 (0.79–1.30)	0.41
No	Yes	No	0.03	2.76 (1.80–4.22)	4.68
Yes	Yes	No	0.01	1.96 (0.97–3.97)	1.25
No	No	Yes	0.06	2.10 (1.50–3.79)	6.44
Yes	No	Yes	0.05	2.73 (1.97–3.79)	8.28
No	Yes	Yes	0.01	7.51 (4.33–13.04)	3.63
Yes	Yes	Yes	0.01	4.97 (2.37–10.40)	1.85
					**26.54**

*Note*: Bold emphasis indicates the total population‐attributable fraction.

Abbreviation: PA, physical activity.

## Discussion

Although sarcopenia and its consequences represent a significant public health problem worldwide, population‐based studies on sarcopenia in developing countries, where the population is rapidly ageing, are still lacking. In this first population‐based study in Vietnam, based on the AWGS 2019 criteria, we show that the prevalence of sarcopenia in men and women aged 50 years and over was 14%. We also identified significant risk factors (e.g., advancing age, being underweight and low physical activity) for sarcopenia. However, the three factors accounted for only 26% of all sarcopenia cases. These findings merit further elaboration.

Our estimated prevalence of sarcopenia is comparable with that observed in other populations. In Thailand and Singapore, sarcopenia was found in between 10% and 14% of the elderly population.^18^, ^19^ A study based on community dwellers in the United States found that 15% of individuals had sarcopenia,^7^ and this prevalence was comparable with estimates from the United Kingdom (using the EWGSOP2 criteria).^20^ Taken together, it appears that the prevalence of sarcopenia in Asian elderly based on the Asian AWGS 2019 criteria is broadly comparable with that of Caucasian populations based on the EWGSOP2 criteria.

We found that being overweight was inversely associated with sarcopenia, suggesting that it may be a protective factor for sarcopenia. This is in line with a previous study in the Caucasian population, which found that overweight individuals had a significantly reduced risk of developing sarcopenia compared with their regular‐weight counterparts.^21^ Moreover, weight gain was associated with a lower risk of sarcopenia.^22^ Thus, our finding further confirms the inverse connection between sarcopenia and being overweight and implies that maintaining a healthy weight is essential for older adults to reduce their risk of sarcopenia.

Our finding supports the idea that high and medium levels of physical activity can delay the development of sarcopenia. Physically active individuals were less likely to transition to sarcopenia.^23^ A previous study identified that a physical activity intervention could improve physical performance in sarcopenic patients.^21^ Conversely, lower habitual physical activity increases the risk of sarcopenia.^24^ In contrast, Volpato et al.^25^ found no relation between sarcopenia and physical activity, but this study was based on bioelectrical impedance rather than DXA. Collectively, our findings and previous observations highlight the importance of physical activity in preventing sarcopenia in the general population.^26^

We found that the proportion of sarcopenia attributable to advancing age, low physical activity and being underweight was modest (26%). This finding suggests that a large proportion of sarcopenia is due to other factors. These ‘residual factors’ may include nutrition and co‐morbidities that we did not ascertain in this study.

Sarcopenia adversely affects the quality of life, metabolic impairment, bone health, reduced functional performance, increased fall risk and increased mortality.^27^ A recent study found a connection between decreased muscular function and an impaired immune system to pathogens.^28^ In an ageing biological system, skeletal muscle may be the primary integrator between immunological senescence and sarcopenia.^29^ Because of their decreased immune function, sarcopenic patients are more likely to become infected and have extended hospital stays and increased medical costs.^30^ Thus, 14% of men and women in the general population have sarcopenia (as observed in this study), implying that sarcopenia is a significant public health problem.

This study has to be interpreted within the context of strengths and limitations. We used state‐of‐the‐art technology (e.g., DXA) to measure lean mass, which ensures the internal validity of the study's finding. The study was based on a large sample size, and the participants were randomly sampled from the general community, which ensures the external validity of the prevalence estimate. However, the participants were drawn from a major city, and the present result may be generalized to something other than rural populations. Like many other epidemiological studies, participants in this study are healthy volunteers compared with those in the general population, and as a result, our estimate could have been biased. Moreover, because this is an observational study, no causal inference could be made on the association between physical activity or being overweight and sarcopenia.

## Conclusions

In summary, based on a population‐based study, we found that 14% of Vietnam adults have sarcopenia, and this estimate is comparable with Caucasian populations. We also found that low physical activity and being underweight were modifiable risk factors for sarcopenia, suggesting that this disease is preventable in the general population.

## Conflict of interest statement

All authors declare no conflicts of interest.
